# Neuroimmune Clearance and EEG Biomarkers: A Unified Model of ASD and Dyslexia

**DOI:** 10.1155/np/6347511

**Published:** 2026-03-31

**Authors:** Günet Eroğlu

**Affiliations:** ^1^ Computer Engineering Department, Engineering and Nature Faculty, Bahçeşehir University, Istanbul, Türkiye, bahcesehir.edu.tr

**Keywords:** ASD, dyslexia, electroencephalography (EEG), meningeal lymphatic system, microglial activation, synaptic pruning

## Abstract

Recent advances in neuroimmunology and cerebrovascular biology have highlighted the important roles of microglial synaptic pruning and the brain’s meningeal lymphatic system in shaping neural circuits during development. Disruptions in one or both of these systems have been reported in neurodevelopmental conditions such as autism spectrum disorder (ASD) and developmental dyslexia. This review synthesizes existing evidence suggesting that impaired meningeal lymphatic clearance may be associated with sustained neuroinflammatory states, which in turn could alter microglial homeostasis and contribute to dysregulated synaptic pruning. We propose a testable theoretical framework linking these cellular and vascular processes to electrophysiological signatures measured by electroencephalography (EEG), while explicitly acknowledging that the majority of available evidence is correlational rather than causal. Reported alterations in EEG frequency bands—such as increased slow‐wave power or disrupted oscillatory coordination—are discussed as potential circuit‐level correlates of underlying neuroimmune dysregulation, rather than definitive mechanistic outcomes. Drawing on findings from both human and animal studies, we outline an integrative conceptual model describing how clearance dysfunction and microglial abnormalities may be associated with patterns of cortical underconnectivity or hyperconnectivity observed in ASD and dyslexia. Rather than establishing causality, this framework aims to generate hypotheses and guide future multimodal investigations combining neuroimmune markers, lymphatic imaging, and electrophysiological measures to evaluate the translational potential of EEG‐informed biomarkers in developmental disorders.

## 1. Introduction

Precise regulation of synapse formation and elimination is fundamental to normal brain development and function. Microglial synaptic pruning plays a central role in this process by selectively removing excess or weak synaptic connections, thereby refining neural circuits during critical developmental windows [1]. In parallel, brain homeostasis is supported by clearance systems that include the glymphatic pathway and the meningeal lymphatic network, which facilitate the removal of metabolic waste products, immune debris, and neurotoxic molecules from the central nervous system (CNS) [2, 3].

Emerging evidence suggests that microglial function and lymphatic clearance are biologically interconnected rather than independent processes. Experimental studies indicate that reduced efficiency of meningeal lymphatic drainage may be associated with elevated neuroinflammatory signaling, which can disrupt microglial homeostasis and compromise synaptic integrity [4]. However, the extent to which lymphatic dysfunction directly alters microglial pruning dynamics in humans remains incompletely understood and is primarily supported by correlational and preclinical data.

Alterations in synaptic pruning and lymphatic clearance have been increasingly implicated in both neurodevelopmental and neurodegenerative conditions. Excessive synaptic elimination has been linked to cortical hypoconnectivity and associated cognitive impairments, whereas insufficient or delayed pruning—frequently discussed in the context of autism spectrum disorder (ASD)—has been associated with local hyperconnectivity and inefficient information processing [5, 6]. Similarly, compromised meningeal lymphatic function may contribute to the accumulation of neurotoxic substances and sustained microglial activation, potentially increasing regional vulnerability in disorders such as developmental dyslexia, particularly within left‐hemisphere language networks [7].

Electroencephalography (EEG) provides a noninvasive means of probing these circuit‐level alterations. Changes in EEG oscillatory activity—such as increased delta and theta power or altered high‐frequency dynamics—have been reported across neurodevelopmental conditions and are commonly interpreted as functional correlates of disrupted synaptic organization or altered network efficiency [8, 9]. Importantly, these electrophysiological patterns should be viewed as indirect indicators of underlying circuit dysfunction rather than direct measures of synaptic pruning or immune activity. Accumulating evidence further suggests that immune and metabolic factors, including vitamin D receptor (VDR) signaling, may modulate both microglial behavior and lymphatic function, providing a potential mechanistic link between immune status, clearance efficiency, and neural circuit maturation.

In this review, we integrate findings from neuroimmunology, electrophysiology, and neuroimaging to propose a conceptual framework linking microglial synaptic regulation and meningeal lymphatic clearance in the context of ASD, developmental dyslexia, and related conditions. Rather than asserting causal relationships, we emphasize converging lines of evidence that support a model in which neuroimmune dysregulation and impaired clearance may be associated with circuit‐level abnormalities detectable by EEG. By framing these interactions as testable hypotheses, this synthesis aims to guide future multimodal studies designed to clarify causal mechanisms and assess whether combined immune, lymphatic, and electrophysiological markers can inform targeted therapeutic strategies for restoring synaptic and cognitive homeostasis.

## 2. Microglial Pruning

Microglia are the resident immune cells of the CNS and play a fundamental role in shaping neuronal networks during brain development and experience‐dependent plasticity. One of their key developmental functions is synaptic pruning, a process through which microglia selectively eliminate or remodel excess, weak, or improperly formed synaptic connections, thereby contributing to the refinement and stabilization of neural circuits [1]. This process is orchestrated by tightly regulated molecular signaling pathways, including the CX3CL1–CX3CR1 axis, TREM2–DAP12 signaling, and components of the complement cascade such as C1q and C3, which tag synapses for removal under physiological conditions [6].

Disruption of these signaling mechanisms has been associated with alterations in synaptic pruning efficiency, which may manifest as either excessive synapse elimination or insufficient pruning. Experimental models suggest that impaired CX3CR1 signaling can delay synaptic maturation and alter social behavior, findings that have been widely discussed in the context of ASD [6]. Conversely, hyperactivation of complement‐mediated pruning has been implicated in disorders characterized by synaptic loss, such as schizophrenia, where excessive synapse elimination and gray matter reductions have been reported [5]. Importantly, these observations largely derive from animal studies or patient‐derived cellular models, and direct evidence linking specific pruning abnormalities to clinical phenotypes in humans remains limited.

Microglial activity is further shaped by a range of extrinsic signals, including pro‐inflammatory cytokines (e.g., interleukin‐1β [IL‐1β] and tumor necrosis factor‐alpha [TNF‐α]), neurotransmitters (e.g., glutamate and ATP), and hormonal factors such as VDR signaling. These influences can shift microglial states along a spectrum from homeostatic to pro‐inflammatory phenotypes, each of which may differentially impact synaptic maintenance and elimination. For example, vitamin D deficiency has been associated with increased pro‐inflammatory microglial signaling and synaptic imbalance in preclinical studies, suggesting a potential modulatory role of metabolic and endocrine factors in neuroimmune regulation of synaptic architecture.

The molecular regulation of microglial pruning therefore represents a critical intersection between immune signaling and synaptic organization. While balanced microglial activity is essential for maintaining optimal connectivity, deviations in either direction—excessive or insufficient pruning—may disrupt network efficiency. Hyperactive microglial responses have been linked to excessive synaptic clearance and network destabilization in neurodegenerative and neuroinflammatory conditions such as multiple sclerosis and Alzheimer’s disease. In contrast, reduced or delayed microglial pruning, as frequently hypothesized in ASD, may permit the persistence of redundant or weak synapses, contributing to local hyperconnectivity and altered sensory processing [6].

Collectively, these findings support a model in which cognitive and neurological function depends on a finely tuned balance between synaptic elimination and preservation. Although substantial progress has been made in identifying molecular pathways that govern microglial pruning, most current evidence remains indirect or model‐based. Future studies integrating molecular, electrophysiological, and imaging approaches in human populations will be essential to clarify how microglial dysregulation contributes to circuit‐level abnormalities across neurodevelopmental disorders.

## 3. Lymphatic Clearance

The glymphatic system facilitates the exchange of cerebrospinal fluid (CSF) and interstitial fluid through perivascular pathways within the brain parenchyma, whereas the meningeal lymphatic system mediates the drainage of macromolecules and immune cells from the subarachnoid space toward cervical lymph nodes [2]. Together, these clearance pathways contribute to the removal of metabolic waste products and neurotoxic proteins, including amyloid‐β and tau, thereby supporting CNS homeostasis. Evidence from animal models and human imaging studies indicates that impaired efficiency of these systems may be associated with the accumulation of neurotoxic substances and increased vulnerability to neuronal dysfunction [3, 4].

Disruption of glymphatic or meningeal lymphatic function has been most extensively studied in the context of neurodegenerative disorders, such as Alzheimer’s disease, where reduced clearance capacity has been linked to protein aggregation and cognitive decline. While these findings support a functional relationship between waste clearance and neural integrity, direct causal links between lymphatic dysfunction and neurodevelopmental pathology remain limited. Nevertheless, regional heterogeneity in lymphatic drainage efficiency may contribute to differential susceptibility of specific neural circuits, potentially influencing cognitive domains that are selectively affected in developmental disorders. Factors such as sleep architecture, vascular dynamics, and circadian regulation have been shown to modulate glymphatic flow, suggesting that clearance efficiency is a dynamic and state‐dependent process rather than a fixed biological trait [2].

Neuroimmune signaling further intersects with lymphatic clearance in maintaining synaptic and circuit stability. Immune dysregulation has been shown to exacerbate synaptic vulnerability and cognitive impairment, particularly under conditions of chronic inflammation [10]. VDR signaling represents one potential modulatory pathway linking immune regulation, microglial activity, and clearance mechanisms. Reduced VDR signaling has been associated with altered neuroimmune responses and impaired removal of neurotoxic molecules in neurodegenerative conditions, although the relevance of these findings to neurodevelopmental disorders remains largely inferential [11].

In the context of ASD and related neurodevelopmental conditions, it has been hypothesized that immune imbalance and altered microglial activity could interact with clearance inefficiency to influence synaptic maintenance. However, empirical evidence directly linking lymphatic dysfunction to synaptic or cognitive outcomes in these populations is currently sparse. Accordingly, potential therapeutic approaches—such as immunomodulatory strategies, dietary interventions, or sleep‐based optimization of glymphatic flow—should be viewed as speculative and hypothesis‐generating rather than established treatments.

Overall, existing findings support a conceptual model in which glymphatic and meningeal lymphatic systems interact with neuroimmune processes to influence brain homeostasis across the lifespan. Clarifying the extent to which these clearance pathways contribute to synaptic regulation and circuit dysfunction in neurodevelopmental disorders will require future studies integrating lymphatic imaging, immune profiling, and functional neurophysiological measures within the same cohorts.

## 4. EEG Biomarkers

EEG provides a noninvasive method for assessing large‐scale neural activity and identifying functional alterations in brain network organization [12]. Across a range of neurological and neurodevelopmental conditions—including ASD, developmental dyslexia, and neurodegenerative diseases—alterations in EEG oscillatory activity, particularly within low‐frequency bands, have been consistently reported [8]. Such slow‐wave activity is generally interpreted as a marker of altered network efficiency or delayed cortical maturation, rather than a direct measure of synaptic pathology.

Changes in EEG oscillations have frequently been discussed in relation to synaptic organization and pruning dynamics. However, EEG signals reflect the summed activity of large neuronal populations and should therefore be regarded as indirect indicators of underlying circuit‐level processes. In neurodevelopmental conditions such as ASD, reduced network coherence—often indexed by measures of global efficiency—has been associated with inefficient information processing and atypical functional connectivity [13]. Whether these electrophysiological features arise from excessive synaptic elimination, insufficient pruning, compensatory network reorganization, or a combination of mechanisms remains unresolved.

A substantial body of literature has documented oscillatory abnormalities in ASD across multiple frequency bands, including delta (1–4 Hz), theta (4–8 Hz), alpha (8–12 Hz), and gamma (>30 Hz). For instance, increased delta and theta power in frontal and posterior cortical regions has been reported in children with ASD, with effect sizes ranging from Cohen’s *d* = 0.8–1.2 in some studies [8, 13]. While these effect sizes are notable, their interpretation must be tempered by methodological considerations, including small sample sizes, developmental heterogeneity, and variability in EEG preprocessing pipelines. Consequently, large effect sizes should not be equated with definitive mechanistic strength.

Alterations in higher‐frequency dynamics have also been described. Misiak et al. [9] reported reduced alpha power and disrupted alpha–gamma phase‐amplitude coupling, findings that have been interpreted as reflecting impaired endogenous control processes and altered large‐scale network coordination. Similarly, abnormalities in cross‐frequency coupling, particularly between alpha and gamma bands, have been observed in frontal and temporal regions in ASD and have been discussed in terms of increased cortical noise or atypical excitation–inhibition balance [14]. These electrophysiological patterns are consistent with models of network dysregulation but do not provide direct evidence for specific cellular mechanisms such as microglial pruning.

Collectively, these findings highlight frequency‐ and region‐specific EEG alterations that may serve as functional correlates of atypical circuit organization in neurodevelopmental disorders. Rather than directing treatment in a prescriptive manner, EEG‐based biomarkers are best conceptualized as candidate indicators for stratifying neural phenotypes and monitoring functional changes in response to experimental or clinical interventions. Their integration with immune markers, neuroimaging, and developmental context will be essential for determining their translational relevance and for testing hypotheses linking neuroimmune processes, synaptic regulation, and large‐scale brain dynamics (Table 1).

**Table 1:** Quantitative summary of EEG abnormalities in ASD and dyslexia.

Disorder	EEG band	Direction of change	Affected regions	Effect size/notes	Sources
ASD	Delta (1–4 Hz)	↑ Increased	Frontal, posterior cortices	*d* = 0.8–1.0	[8, 13]
ASD	Theta (4–8 Hz)	↑ Increased	Frontal, right posterior cortex	*d* = 1.2	[13]
ASD	Alpha (8–12 Hz)	↓ Decreased	Frontal, parietal, temporal	Hypoinhibition; PAC disrupted	[9, 15]
ASD	Gamma (>30 Hz)	↑ Increased	Midline, occipital	Cortical noise; abnormal PAC	[14]

An expanding body of research indicates that neuroimmune signaling is associated with measurable changes in brain oscillatory dynamics. Experimental and clinical studies have reported links between cytokine‐mediated immune activation and alterations in EEG activity, particularly within low‐frequency bands such as delta (1–4 Hz) and theta (4–8 Hz), suggesting that immune dysregulation may influence large‐scale neural synchrony and functional connectivity [16]. Similarly, systemic inflammation has been associated with EEG slowing and desynchronization, pointing to a functional coupling between peripheral immune signals and cortical excitability. Early‐life adversity and sustained pro‐inflammatory signaling have also been implicated in delayed or altered electrophysiological maturation, further supporting an association between immune status and EEG‐based biomarkers [17].

From a mechanistic perspective, preclinical studies have proposed that excessive microglial activation can alter synaptic organization, particularly through increased elimination of excitatory synapses within cortical and thalamocortical circuits [5, 6]. Such synaptic alterations have been hypothesized to reduce effective synaptic drive and network integration, potentially giving rise to compensatory changes in intrinsic neuronal properties and network oscillations. For example, animal models exhibiting excessive synaptic pruning frequently display increased slow‐wave activity and reduced high‐frequency coherence, especially in sensory cortices [1]. However, whether similar mechanisms operate in the human brain—and to what extent they contribute to EEG abnormalities observed in neurodevelopmental disorders—remains uncertain.

These observations have motivated conceptual models linking cellular‐level synaptic alterations to macroscopic electrophysiological patterns. Importantly, microglial pruning does not occur in isolation but is embedded within broader homeostatic systems, including glymphatic and meningeal lymphatic clearance pathways. Activated microglia release pro‐inflammatory cytokines (e.g., IL‐1β, TNF‐α, and IL‐6) and reactive oxygen species, which can alter the perivascular and interstitial environments and increase the burden of metabolic byproducts requiring efficient clearance [2, 3]. Impairments in these clearance systems—attributed to factors such as sleep disruption, genetic susceptibility, or vascular alterations—have been associated with the accumulation of neurotoxic molecules, including amyloid‐β and tau, and with sustained inflammatory signaling [4, 11].

Together, these processes have been proposed to form a self‐reinforcing cycle in which impaired clearance may amplify neuroinflammatory load, thereby sustaining microglial dysregulation and atypical synaptic remodeling. Microglia are also sensitive to ionic and osmotic changes influenced by CSF dynamics, raising the possibility that altered waste outflow could indirectly modulate pruning behavior [2]. In neurodevelopmental conditions such as ASD and developmental dyslexia, immune alterations, microglial dysfunction, and atypical clearance processes have been reported to co‐occur; however, their temporal ordering and causal interdependence remain to be established [18, 19].

Despite the conceptual coherence of this integrative framework, the existing literature remains predominantly correlational. Most studies infer relationships between immune activity and electrophysiological changes based on parallel observations—such as elevated cytokine levels alongside atypical EEG rhythms—rather than through direct experimental manipulation or longitudinal causal inference. Consequently, it remains unclear whether increased delta and theta power in ASD and dyslexia primarily reflects altered synaptic pruning, metabolic or vascular dysfunction, neurovascular uncoupling, compensatory network reorganization, or a combination of these mechanisms.

Moreover, the specific contributions of glymphatic and meningeal lymphatic systems to neurodevelopmental pathology are still insufficiently characterized in humans. Although these clearance pathways are increasingly recognized as critical regulators of brain homeostasis, few studies have employed noninvasive imaging techniques—such as diffusion‐based MRI, dynamic contrast‐enhanced MRI, or near‐infrared spectroscopy (NIRS)—to assess their function during early development. As a result, the proposed feedback loop linking clearance impairment, inflammation, and synaptic dysregulation should be regarded as a hypothesis‐generating model rather than an established biological pathway.

To advance beyond associative interpretations, future research will require integrative, multimodal study designs capable of simultaneously assessing immune markers, lymphatic function, and EEG dynamics within the same developmental cohorts. Such approaches are essential for determining whether neuroimmune dysfunction plays a causal role in shaping circuit‐level abnormalities observed in disorders such as ASD and dyslexia and for evaluating the translational utility of EEG‐informed neuroimmune biomarkers.

## 5. ASD

ASD is characterized by region‐specific alterations in brain connectivity and immune signaling. Neuroimaging studies have consistently reported atypical functional activity and differences in gray matter volume in regions including the right frontal cortex, superior temporal sulcus, and motor‐related areas, which are implicated in executive functioning, social cognition, and motor planning [20]. These structural and functional findings have been discussed alongside evidence of altered synaptic development and immune regulation, although the precise mechanisms linking these processes remain incompletely defined.

Accumulating research suggests that neuroimmune factors and clearance‐related processes may contribute to regional vulnerability in ASD. The deposition of neurotoxic proteins or pro‐inflammatory mediators within perivascular and meningeal lymphatic compartments has been associated with altered synaptic remodeling and microglial surveillance in experimental models [2, 3]. The right frontal cortex, which undergoes prolonged maturation and exhibits heightened plasticity during early childhood, has been proposed as a candidate region that may be particularly sensitive to disruptions in immune homeostasis and waste clearance. Factors such as sleep disturbances—commonly reported in individuals with ASD—may further influence clearance efficiency and local inflammatory tone, although direct evidence in pediatric populations remains limited [4].

Electrophysiological studies in ASD have frequently reported increased low‐frequency (delta and theta) power and reduced alpha‐band coherence, particularly over frontal regions [13, 15]. These EEG features are generally interpreted as markers of delayed or inefficient network organization rather than as direct indicators of specific cellular mechanisms. Within a conceptual framework, such oscillatory patterns may reflect the convergence of anatomical vulnerability, altered immune signaling, and atypical synaptic refinement; however, alternative explanations—including compensatory network reorganization or developmental heterogeneity—cannot be excluded.

Structural and functional abnormalities involving frontal and motor cortices are among the most consistently replicated findings in ASD [20] and are thought to contribute to impairments in executive control and motor coordination. Experimental studies have proposed that insufficient glymphatic clearance, delayed synaptic maturation, and reduced or altered pruning may increase regional susceptibility to network inefficiency [2, 4, 6]. Nevertheless, the relevance of these mechanisms to human ASD remains largely inferential.

Accordingly, potential intervention strategies aimed at modulating immune signaling or enhancing clearance pathways should be regarded as hypothesis‐generating rather than established treatments. Future studies integrating neuroimaging, immune profiling, lymphatic assessment, and EEG within the same cohorts will be essential for determining whether these processes play a causal role in shaping ASD‐related circuit abnormalities.

Aberrant synaptic development and neuroinflammatory processes have been hypothesized to contribute to the characteristic “U‐shaped” EEG power profile reported in some individuals with ASD. This profile is typically described as a relative increase in low‐frequency activity alongside elevated high‐frequency power, accompanied by reductions in mid‐frequency bands. Importantly, this pattern should be regarded as a descriptive electrophysiological phenotype rather than a direct indicator of specific cellular mechanisms.

Low‐frequency abnormalities, particularly increased delta (1–4 Hz) and theta (4–8 Hz) power, have been repeatedly observed in ASD, most prominently over frontal and right posterior cortical regions [13]. These oscillatory features are often interpreted as markers of delayed or inefficient network organization and may reflect compensatory activity within circuits exhibiting altered connectivity. Within a theoretical framework, an imbalance between excitatory and inhibitory signaling—potentially influenced by chronic neuroinflammatory states—has been proposed to contribute to an increased ratio of low‐ to high‐frequency power [7]. However, such interpretations remain inferential and do not establish causality.

Mid‐frequency alterations, particularly reduced alpha (8–12 Hz) power across frontal, occipital, parietal, and temporal regions, have also been reported in ASD [15]. Decreased alpha activity is commonly associated with reduced inhibitory control and atypical large‐scale coordination. While incomplete or altered synaptic maturation has been proposed as one contributing factor, alpha‐band reductions may also arise from broader disruptions in network timing, task engagement, or developmental heterogeneity [9]. Elevated levels of pro‐inflammatory cytokines, such as TNF‐α and IL‐6, have been associated with altered synaptic homeostasis and may further modulate alpha‐band dynamics, although direct links between cytokine signaling and oscillatory changes in humans remain limited [21].

Increased beta (13–30 Hz) and gamma (>30 Hz) power has been reported in occipital, parietal, and midline regions in subsets of individuals with ASD, findings often discussed in terms of local hyperconnectivity or increased cortical “noise.” Within hypothetical models, insufficient synaptic clearance or altered excitation–inhibition balance could permit excessive high‐frequency activity. Nonetheless, gamma‐band abnormalities are multifactorial and may also reflect deficient GABAergic inhibition, altered interneuron function, or compensatory network reorganization, rather than neuroinflammatory pruning failure alone.

Taken together, the “U‐shaped” EEG profile has been proposed as a functional manifestation of excitation–inhibition imbalance at the network level. Preclinical studies suggest that altered microglial signaling—such as reduced CX3CR1 activity or elevated inflammatory cytokines—can interfere with synaptic maturation and pruning [6, 7]. However, EEG findings in ASD are heterogeneous, with mixed or developmentally variable patterns reported across age groups, ASD subtypes, and comorbid conditions. This variability underscores the need for stratified analyses rather than uniform mechanistic interpretations.

Despite the conceptual appeal of neuroimmune models linking microglial dysfunction to EEG abnormalities, it remains unclear whether oscillatory alterations in ASD directly reflect immune signaling or arise secondarily from large‐scale network reorganization. For example, gamma hyperactivity may result from both hyperconnectivity and reduced inhibitory control, independent of inflammatory mechanisms. Longitudinal and multimodal studies combining EEG, immune profiling, and lymphatic or vascular imaging will be essential to clarify the temporal ordering and causal relationships among these processes.

Overall, accumulating evidence supports an association between altered synaptic development, neuroimmune dysregulation, and atypical EEG patterns in ASD. However, regional microglial activity may vary across the cortex, leading to heterogeneous patterns of over or underpruning that differentially affect network efficiency and coherence. Accordingly, EEG abnormalities in ASD are best interpreted as functional correlates of complex developmental processes rather than as direct readouts of specific cellular pathologies.

## 6. Dyslexia

Developmental dyslexia is increasingly conceptualized not only as a disorder of phonological processing but also as a neurodevelopmental condition involving regionally specific alterations in neural circuit organization. These alterations predominantly affect left‐hemisphere language networks responsible for orthographic mapping and phonological decoding [19]. In contrast to ASD, which is often characterized by widespread alterations in cortical connectivity, functional disruptions in dyslexia appear to be more spatially circumscribed and task‐specific. In certain dyslexia subtypes, impairments have also been proposed within visual processing pathways, particularly those involving the magnocellular system, although this framework remains debated [22].

Electrophysiological studies have consistently reported increased theta‐band (4–8 Hz) power over the left temporoparietal cortex and reduced suppression of alpha‐band (8–12 Hz) activity during phonological and reading‐related tasks in individuals with dyslexia [23]. These oscillatory patterns are commonly interpreted as reflecting impaired top–down control and delayed thalamocortical or cortico‐cortical coordination, rather than as direct indicators of specific synaptic mechanisms. Emerging evidence further suggests that dyslexia may be associated with alterations in beta‐band connectivity, pointing toward broader inefficiencies in executive and cognitive control networks.

At the level of immune signaling, several studies have reported elevated peripheral inflammatory markers—including C‐reactive protein (CRP), IL‐1β, and TNF‐α—in subsets of children with dyslexia [24]. Given the established role of these cytokines in modulating microglial activation and synaptic plasticity, such findings raise the possibility that neuroimmune factors could influence synaptic development within language‐related circuits. However, the extent to which peripheral immune markers reflect CNS immune activity in dyslexia remains unclear [25].

Importantly, direct evidence linking dyslexia to microglial dysfunction or abnormal synaptic pruning is currently limited. Unlike ASD—where impaired CX3CR1 signaling and altered microglial pruning have been demonstrated in animal and cellular models—dyslexia has not yet been systematically investigated using microglia‐specific approaches such as transcriptomic profiling, microglia‐targeted PET imaging, or postmortem histological analyses. Consequently, associations between EEG abnormalities and putative microglial states in dyslexia should be regarded as correlational and hypothesis‐generating rather than mechanistically established.

This gap in empirical evidence highlights a critical direction for future research. Integrative studies combining EEG‐based oscillatory measures with immune profiling (e.g., cytokine panels or VDR signaling) and advanced neuroimaging modalities—such as PET, MRI, or NIRS—will be essential for evaluating whether immune dysregulation or delayed synaptic refinement contributes causally to the dyslexic phenotype. Such multimodal approaches may also clarify whether the frequently observed reliance on right‐hemisphere compensatory networks and reduced specialization of the left temporoparietal cortex reflect underlying neuroimmune influences or alternative developmental mechanisms.

In summary, dyslexia may arise from the interaction of regionally confined network inefficiencies, delayed or altered synaptic maturation, and subtle neuroimmune imbalances. Although neuroimmune disruption appears to be more pervasive in ASD, the accumulating electrophysiological and immunological evidence in dyslexia underscores the need for equally rigorous mechanistic investigations. A clearer understanding of these processes may eventually inform EEG‐guided, developmentally timed interventions, while recognizing that such applications remain speculative pending causal validation.

## 7. Discussion

Although microglial synaptic pruning and lymphatic clearance have each been investigated in the context of neurodevelopment, their combined influence on large‐scale brain network stability remains insufficiently characterized. This review aimed to synthesize findings across neuroimmunology, electrophysiology, and cerebrovascular biology to outline an integrative framework linking these processes, while recognizing the current limitations of the evidence base.

One major challenge in this field is the considerable variability across experimental models, tissue types, and developmental stages. Studies of ASD, particularly in animal models, have yielded heterogeneous and sometimes contradictory findings regarding the extent, timing, and regional specificity of microglial pruning alterations [6]. These discrepancies likely reflect differences in brain region examined, developmental window, experimental manipulation, and genetic background, as well as the marked clinical heterogeneity of ASD itself. As a result, generalizing pruning‐related mechanisms across the autism spectrum remains difficult, underscoring the need for systematic cross‐model and cross‐species validation.

The relationship between synaptic pruning abnormalities and EEG oscillatory patterns represents another unresolved issue. Although increased delta and theta power has been consistently reported in both ASD and developmental dyslexia, it remains unclear whether these oscillatory features directly reflect altered synaptic pruning, compensatory network reorganization, delayed maturation, or broader neurovascular and metabolic factors. The lack of studies simultaneously assessing cellular, immune, and electrophysiological markers within the same individuals further limits causal inference. Multimodal approaches that integrate EEG with immune profiling and measures of synaptic or circuit integrity will therefore be essential for disentangling these possibilities.

Knowledge gaps are particularly pronounced with respect to the human glymphatic and meningeal lymphatic systems in neurodevelopment. While preclinical evidence supports a role for clearance pathways in maintaining cognitive integrity, standardized and developmentally appropriate methods for assessing lymphatic function in humans—especially in pediatric populations—are still lacking. The absence of validated noninvasive biomarkers constrains the translational relevance of animal findings and complicates efforts to test clearance‐based hypotheses in developmental disorders. Advances in neuroimaging techniques, such as diffusion‐based MRI, dynamic contrast‐enhanced MRI, or NIRS, may help address these limitations in future studies.

**Figure 1:**
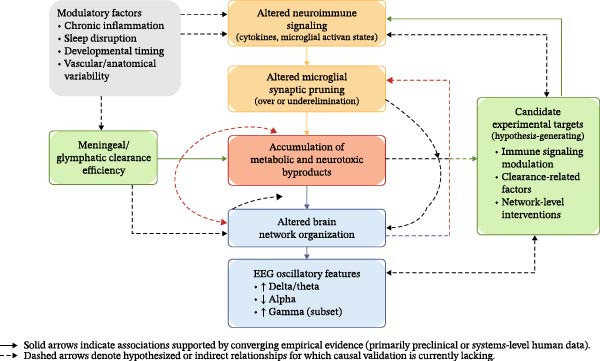
Conceptual model illustrating the relationships among microglial synaptic pruning, lymphatic clearance dysfunction, neuroinflammation, and EEG biomarkers. Aberrant microglial activation—either excessive or insufficient—leads to disrupted synaptic pruning. This is often driven by chronic neuroinflammation and is exacerbated by impaired glymphatic and meningeal lymphatic drainage, which fail to clear metabolic waste and neurotoxic proteins. These combined disruptions alter neuronal connectivity, resulting in abnormal EEG patterns (e.g., enhanced slow‐wave activity, decreased alpha coherence, and increased gamma noise), as observed in conditions such as ASD and dyslexia. The model highlights potential therapeutic targets: restoring immune balance, enhancing waste clearance, and normalizing brain oscillations.

More broadly, the current literature is characterized by fragmentation across disciplinary boundaries. To date, no published study has concurrently evaluated brain oscillatory dynamics, lymphatic clearance function, and peripheral immune status within the same neurodevelopmental cohort. This lack of integrative data hampers the construction of cohesive mechanistic models linking neuroimmune dysregulation to electrophysiological alterations. Progress toward clinically meaningful translation will require coordinated, interdisciplinary study designs capable of bridging molecular, systems‐level, and functional perspectives (Figure 1).

## 8. Future Research

To advance our understanding of neurodevelopmental disorders and their associated neuroimmune mechanisms, the following interdisciplinary research priorities are proposed:

### 8.1. Multimodality Integration

Combining EEG, fMRI, lymphatic imaging, and immunological biomarkers within the same cohort can clarify how neural activity, immune signaling, and waste clearance interact dynamically. This integrative approach is essential for disentangling the complex biological cascades underlying cognitive and behavioral outcomes in conditions such as ASD and dyslexia.

### 8.2. Biomarker Development

There is a critical need to identify and validate EEG‐based biomarkers that reflect synaptic pruning efficiency and lymphatic clearance capacity. These should be cross‐referenced with peripheral and central biochemical markers—such as CSF cytokines or serum levels of vitamin D—to ensure clinical applicability and biological relevance.

### 8.3. Therapeutic Exploration

Early‐phase clinical trials are needed to assess interventions targeting microglial modulation (e.g., vitamin D supplementation and IL‐10 agonists) and enhancement of glymphatic function (e.g., sleep‐promoting strategies and lymphatic flow enhancers). Given their interdependent roles in synaptic remodeling and metabolic clearance, targeting both systems may offer synergistic therapeutic benefits.

### 8.4. Oscillatory Signatures and Behavioral Correlates

Changes in EEG frequency power—such as increased delta/theta or reduced alpha coherence—may reflect underlying synaptic and immune dysfunctions. These oscillatory signatures have the potential to serve as mechanistic biomarkers that bridge physiological abnormalities and clinical symptoms, aiding both diagnosis and treatment personalization.

### 8.5. Closing the Causality Gap

To move beyond correlational interpretations, future research must overcome current methodological fragmentation by integrating EEG, PET, and lymphatic imaging within the same developmental cohorts. Such studies are crucial for establishing direct causal links among neuroimmune activity, oscillatory dynamics, and cognitive–behavioral outcomes.

## 9. Conclusions

This review presents a conceptual framework in which microglial synaptic regulation and meningeal/glymphatic clearance are considered functionally interconnected components of brain homeostasis during neurodevelopment, rather than independent or parallel processes. By integrating findings from neuroimmunology, neuroimaging, and electrophysiological studies, we synthesize evidence suggesting that alterations in immune signaling, synaptic refinement, and waste clearance are frequently observed alongside atypical network organization. Importantly, these associations do not establish causality but highlight converging biological domains that warrant integrated investigation.

Despite their distinct clinical presentations, ASD and developmental dyslexia share overlapping features, including alterations in immune‐related pathways, regionally specific synaptic irregularities, and characteristic EEG patterns. These commonalities support the exploration of cross‐diagnostic frameworks focused on neuroimmune and network‐level processes, while recognizing substantial heterogeneity across individuals and developmental stages. Establishing causal relationships among these factors will require longitudinal, multimodal, and stratified study designs capable of resolving temporal dynamics and individual variability.

Rather than proposing established therapeutic strategies, the framework outlined here is intended to generate testable hypotheses regarding how immune modulation, clearance‐related processes, and large‐scale brain dynamics may interact during development. EEG‐based measures, in combination with immune profiling and neuroimaging, may offer a functional perspective for characterizing neural phenotypes and monitoring developmental trajectories. However, the translational relevance of such approaches remains to be determined through rigorous empirical validation.

In conclusion, advancing neurodevelopmental research beyond associative models will depend on integrative methodologies that bridge cellular, systems‐level, and functional measures. By framing neuroimmune clearance and oscillatory dynamics as interacting but unproven contributors to circuit organization, this review aims to provide a cautious yet coherent foundation for future interdisciplinary research into the biological underpinnings of ASD, dyslexia, and related conditions.

## Funding

The authors have nothing to report.

## Conflicts of Interest

The author declares no conflicts of interest.

## Data Availability

The data that support the findings of this study are available upon request from the corresponding author. The data are not publicly available due to privacy or ethical restrictions.
